# 

**Published:** 1877-11

**Authors:** 


The following pamphlets have been received:

Report on Dermatology, Syphilis, and other Exanthemata. By Lunsford P. Yandall, Jr., M.D., Prof. of Therapeutics and Clinical Medicine, University of Louisville. Reprint from the transactions of the Kentucky State Medical Society, 1877.

The Cause and Geographical Distribution of Calculous Diseases. By Claudius H. Mastin, M.D., of Mobile, Ala. Extracted from the transactions of the International Medical Congress, Philadelphia, September, 1876. Philadelphia, 1877.

Address on Medical Biography. Delivered before the International Medical Congress, September, 1876. By J. M. Toner, M.D., Washington, D. C. Philadelphia, 1876.

Strictures of the Urethra; When and How shall we Perform Internal Urethrotomy? By Claudius H. Mastin, M.D., LL.D., Mobile, Ala. From November No. Richmond and Louisville Medical Journal, Louisville, 1877.

Treatment of Diphtheria. By E. N. Chapman, A.M., M.D., late Professor of Obstetrics, L. I. College Hospital. Baker, Jones & Co., Buffalo, 1877.

Poisonous effects of Cyanide of Potassium. By Joseph Jones, M.D., Professor of Chemistry and Clinical Medicine, Medical Department University of Louisiana, etc. From the *New Orleans Medical and Surgical Journal*, May, 1877.

The Discovery of Anæsthesia. By J. Marion Sims, M.D., Reprint from *Virginia Medical Monthly* ,May, 1877.

